# A Flow Sensor-Based Suction-Index Control Strategy for Rotary Left Ventricular Assist Devices

**DOI:** 10.3390/s21206890

**Published:** 2021-10-18

**Authors:** Lixue Liang, Kairong Qin, Ayman S. El-Baz, Thomas J. Roussel, Palaniappan Sethu, Guruprasad A. Giridharan, Yu Wang

**Affiliations:** 1 School of Mechanical Engineering, Dalian University of Technology, No. 2 Linggong Road, Ganjingzi District, Dalian 116024, China; lixueliang@mail.dlut.edu.cn; 2 School of Optoelectronic Engineering and Instrumentation Science, Dalian University of Technology, No. 2 Linggong Road, Ganjingzi District, Dalian 116024, China; krqin@dlut.edu.cn; 3 Department of Bioengineering, University of Louisville, Louisville, KY 40292, USA; ayman.elbaz@louisville.edu (A.S.E.-B.); thomas.roussel@louisville.edu (T.J.R.); guruprasad.giridharan@louisville.edu (G.A.G.); 4 Department of Biomedical Engineering, School of Engineering, University of Alabama at Birmingham, 1075 13th St. S., Birmingham, AL 35294, USA; psethu@uabmc.edu

**Keywords:** left ventricular assist devices, sensor-based control, pump independent, suction index, physiological perfusion, suction prevention

## Abstract

Rotary left ventricular assist devices (LVAD) have emerged as a long-term treatment option for patients with advanced heart failure. LVADs need to maintain sufficient physiological perfusion while avoiding left ventricular myocardial damage due to suction at the LVAD inlet. To achieve these objectives, a control algorithm that utilizes a calculated suction index from measured pump flow (SIMPF) is proposed. This algorithm maintained a reference, user-defined SIMPF value, and was evaluated using an in silico model of the human circulatory system coupled to an axial or mixed flow LVAD with 5–10% uniformly distributed measurement noise added to flow sensors. Efficacy of the SIMPF algorithm was compared to a constant pump speed control strategy currently used clinically, and control algorithms proposed in the literature including differential pump speed control, left ventricular end-diastolic pressure control, mean aortic pressure control, and differential pressure control during (1) rest and exercise states; (2) rapid, eight-fold augmentation of pulmonary vascular resistance for (1); and (3) rapid change in physiologic states between rest and exercise. Maintaining SIMPF simultaneously provided sufficient physiological perfusion and avoided ventricular suction. Performance of the SIMPF algorithm was superior to the compared control strategies for both types of LVAD, demonstrating pump independence of the SIMPF algorithm.

## 1. Introduction

Heart failure (HF) is a highly prevalent disease and a leading cause of mortality in the world, with approximately 2% of adults suffering from HF worldwide [1]. The most effective treatment for advanced HF is heart transplantation [2], but due to the limited number of available donor hearts, only a few thousand patients in the world receive heart transplantation every year, with more than 20% of waitlisted patients perishing before a donor heart becomes available [3]. Rotary left ventricular assist devices (LVAD), surgically implantable mechanical blood pumps, have been increasingly utilized as a long-term treatment for advanced HF patients. The pump inlet is attached to the apex of the left ventricle (LV) and the pump outlet is anastomosed to the aorta. The LVAD pumps blood from the LV to the aorta, alleviating the workload of the native heart, thereby serving as a bridge to transplantation or destination therapy [4,5]. Rotary LVADs have effectively replaced pulsatile LVADs since rotary LVADs are mechanically simpler, smaller, lighter weight, and have higher operating efficiencies. Additionally, rotary LVADs are more durable, showing improved survival rates compared to pulsatile LVADs [6,7].

LVADs must generate sufficient physiological perfusion as insufficient pump flow rates can lead to hypoperfusion, pulmonary edema, and volume overload of the native LV. Simultaneously, LVADs must also avoid suction due to over pumping, which can result in severe LV decompression and/or LVAD inflow obstruction. Suction events can trigger pump flow stoppage, LV collapse, myocardial damage, or induce myocardial arrhythmias, each of which may lead to potentially life-threatening events or death [8]. Pulsatile LVADs have low risk of LV suction due to phasic filling, which results in a higher preload sensitivity. In contrast, suction occurs commonly during the rotary LVAD support due to its lower preload sensitivity [9,10]. For example, 15 out of 19 patients with rotary LVAD support experienced suction events with 13 suction events per 1000 min of support [11]. Patients with rotary LVADs are also prone to suction during Valsalva maneuver, coughing, hypovolemia, and transient reduction in cardiac return [12]. Therefore, avoiding LV suction while providing adequate pump-augmented cardiac output (CO) during various levels of activity is critical for patients on LVAD support.

Suction detection algorithms based on a variety of pump signals for rotary LVADs have been proposed [13,14,15,16], however, these algorithms only detect suction events after they have occurred, which results in myocardial damage. Many physiological control strategies have also been developed that aim to reduce LV suction events [17,18,19,20,21,22], but some of these require the measurement of ventricular pressure and/or volume, which require sensors that are in contact with blood, and thus susceptible to thrombosis or failure, while others may not be able to generate sufficient perfusion during varying physiological conditions. Our group and others have developed sensorless algorithms [23,24,25,26] with model-based parameter estimation strategies. However, the performance of these algorithms may be adversely affected by changes in blood viscosity, friction forces, and device inertia [27]. Recently, non-model-based, sensorless control algorithms have been proposed [27,28], but the performance of the differential pump speed (Δ*RPM*) controller may be degraded by increased levels of measurement noise or with rapidly changing ventricular contractilities. Furthermore, obtaining the suction index (*SI*) from the pump speed (PS) cannot be pump-independent. Constant parameter-based control strategies by maintaining constant pump speed (CPS), LV end-diastolic pressure (LVEDP), mean aortic pressure (MAoP), and pressure head (Δ*P*) across the LVAD have also been proposed in the literature [29,30,31]. While these control algorithms can be effective in a limited set of conditions, they may not be adequate with changing physiologic demand conditions.

In this paper, a new pump-independent, flow sensor based control algorithm is proposed. In contrast to pressure and volume sensors, ultrasonic flow sensors are implanted outside the pump outflow graft and do not come in contact with blood. Flow sensors have been clinically implanted in patients with the HeartAssist 5 LVAD [32]. In this manuscript, we utilize the measured pump flow (PF) signal (e.g., SIMPF control) with 5% and 10% normally distributed noise added to the original signal. The feasibility of the control algorithm to sense the adequate level of perfusion and avoid suction was tested for two different types of rotary LVADs to test for pump independence. Performance of the proposed SIMPF control strategy was evaluated in silico under different simulated conditions and compared to other control algorithms that are clinically used or previously reported in the literature.

## 2. Methods

### 2.1. Modeling of the Biventricular Cardiovascular System

In this study, a validated and published model of a biventricular cardiovascular system was used to develop the SIMPF control algorithm. The lumped parameter model has previously been used for testing control algorithms, timing algorithms, and fault detection algorithms with different types of LVADs [27,33,34]. Four valves and twelve lumped parameter blocks constitute the model [28]. Amongst these blocks, nonlinear active elements include the left and right atrium and ventricles because their values of compliance were time-varying, and the other blocks had time-invariant compliance values. Each block was represented using the differential equation that described the rate of change of volume (*V*) as a function of resistance (*R*), and compliance (*C*), as follows:

(1) $$\frac{dV_{n}}{dt}=F_{n}^{in}-F_{n}^{out}$$

(2) $$\frac{dV_{n}}{dt}=\frac{V_{n-1}}{C_{n-1}R_{n-1}}-\frac{V_{n}}{C_{n}}\left(\frac{1}{R_{n-1}}+\frac{1}{R_{n}}\right)+\frac{V_{n+1}}{C_{n+1}R_{n}}$$

where in block *n*, *dV_n_*/*dt* is the rate of volumetric change; *F^in^* is blood flowing into the block *n*; and *F^out^* is blood flowing out of the block *n*. A model of a rotary LVAD, which was an axial flow pump (AFP) or Deltastream mixed flow pump (DP2), was incorporated into this circulatory system model.

### 2.2. The Rotary LVAD Model

The model of the axial flow rotary LVAD is described by the following two ordinary differential equations:

(3) $$J\frac{d\omega }{dt}=\frac{3}{2}K_{B}I-B\omega -a_{0}\omega^{3}-a_{1}F_{p}\omega^{2}$$

(4) $$\frac{dV_{n}}{dt}=\frac{V_{n-1}}{C_{n-1}R_{n-1}}-\frac{V_{n}}{C_{n}}\left(\frac{1}{R_{n-1}}+\frac{1}{R_{n}}\right)+\frac{V_{n+1}}{C_{n+1}R_{n}}$$

where the various model parameters (*J*, *ω*, *K_B_*, *I*, *B*, *a_0_*, *a_1_*, *F_p_*, *b_0_*, *b_1_*, and *b_2_*) in Equations (3) and (4) and their associated values can be found in [35,36]. *ω* is the LVAD pump speed, the pump current (control variable) is represented by *I*, and *F_p_* is the LVAD pump flow.

A mixed flow LVAD (DP2) model was also simulated in this study to demonstrate the pump independence of the SIMPF control algorithm. The model of DP2 was developed by Petrou et al. [37]:

(5) $$\frac{d\omega }{dt}=\frac{1}{J\left(\omega \right)}\left(k_{T}I-g_{1}\left(\omega \right)+g_{2}\omega -g_{3}\omega^{2}-g_{4}F_{p}\omega \right)$$

(6) $$\frac{dF_{p}}{dt}=-\frac{1}{F}\left(-\Delta P+f_{1}\omega^{2}-f_{2}F_{P}-f_{3}F_{p}^{2}\right)$$

The various model parameters (*J*(*ω*), *k_T_*, *g_1_*(*ω*), *g_2_*, *g_3_*, *g_4_*, *f_1_*, *f_2_*, *f_3_*) in Equations (5) and (6) and their associated values can be found in [37]. Either AFP or DP2 was incorporated into the biventricular cardiovascular model to remove volume from the LV and to add volume to the aorta.

### 2.3. SIMPF Control Strategy

The SIMPF control strategy was developed to keep a single fixed setpoint and provide adequate COs for the circulatory system under varying physiological conditions, while preventing LV suction. In order to achieve this proposed control algorithm, the real-time *SI* was extracted with a sampling rate of 100 Hz and a moving window of 5 s [12,14]. The window was recalculated every 0.1 s:

(7) $$SI=\frac{max\left[\frac{d\left(PF\right)}{dt}\right]-min\left[\frac{d\left(PF\right)}{dt}\right]}{\max\left(PF\right)}$$

**Figure 1:**
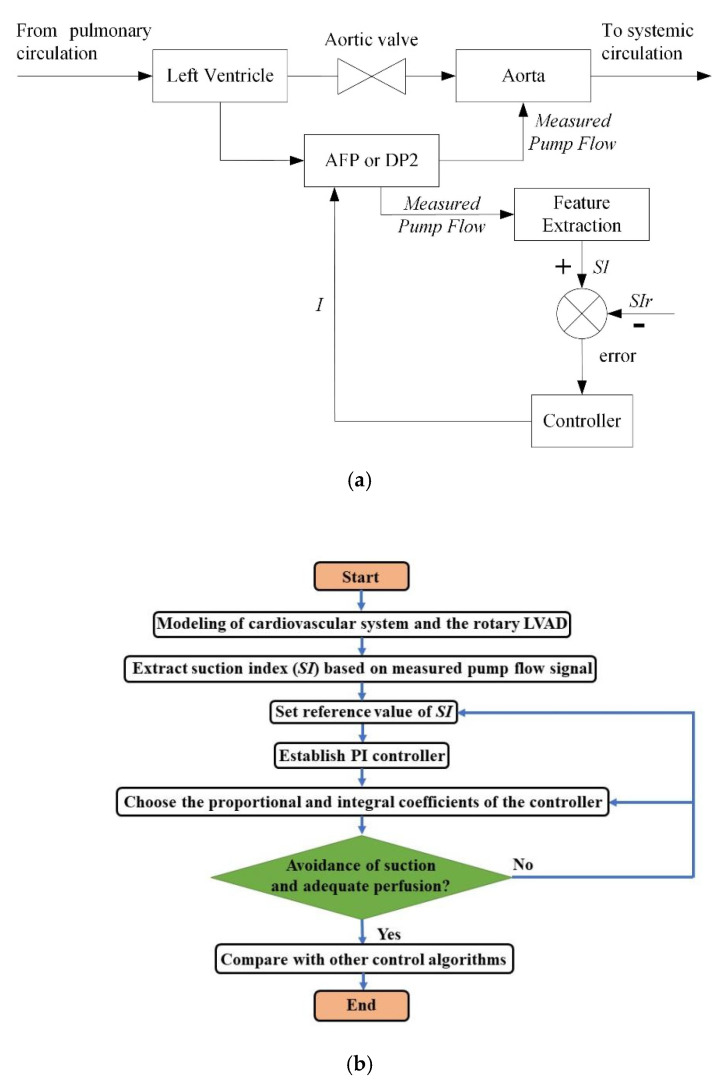
(**a**) Diagrammatic drawing of the proposed suction-index based measured pump flow (SIMPF) control algorithm. The measured pump flow (PF) signals were used to calculate suction index (*SI*) and fed to the PI controller with the reference *SI* (*SI_r_*) for axial flow pump (AFP) and Deltastream mixed flow pump (DP2), respectively, which were surgically implanted from the left ventricular to the aorta. (**b**) Flowchart of the proposed *SI* control method.

In this study, *PF* was measured using the flow sensor including 5% and 10% uniformly distributed measurement noise, and the implementation of the SIMPF control strategy depended on a gain scheduled PI controller. The following control law was used to update the pump current:

(8) $$I=K_{P}\left(SI-SI_{r}\right)+K_{I}\int_{0}^{t}\left(SI-SI_{r}\right)dt$$

where the *SI* setpoint is represented by *SI_r_*, and the proportional and integral coefficients are represented by *K_P_* and *K_I_*, respectively. These parameters can be determined *a priori* [38] and were unchanged during all test conditions in both pumps. In this study, the controller of AFP used 9, 0.07, and 0.014 for *SI_r_*, *K_P_*, and *K_I_*, respectively, while the controller of DP2 set 5, 0.15, and 0.03 for *SI_r_*, *K_P_*, and *K_I_*, respectively. Figure 1 shows the schematic of the proposed SIMPF control strategy and the related flowchart, respectively.

### 2.4. Comparison with the Other Control Strategies

The SIMPF control strategy was compared to previously reported control strategies. (1) sensorless Δ*RPM* control that kept the actual Δ*RPM* above a fixed setpoint, Δ*RPMr*. The actual Δ*RPM* was obtained using the difference between the maximum and minimum PS as described in [27]. The reference Δ*RPM_r_* was set to 800 RPM for AFP to satisfy the physiological demands during rest (PF was 5 L/min), and *K_P_* and *K_I_* were 0.00025 and 0.00005, respectively. For DP2, Δ*RPM_r_* was set to 150 RPM, *K_P_* and *K_I_* were 0.0004 and 0.00008, respectively. (2) Maintain a CPS [28]. The setpoint of PS was 10,452 RPM selected for AFP to provide sufficient physiological perfusion at rest, and *K_P_* and *K_I_* were 0.003 and 0.0006, respectively. In addition, the PS setpoint was 4338 RPM, and *K_P_* and *K_I_* were 0.006 and 0.0012 for DP2, respectively. This sensorless control method was regarded as CPS control, the current clinical standard. (3) Control the average Δ*P* from the LV to aorta across an LVAD, Δ*P* control. Δ*P* can be estimated using pump speed measurements with 2% noise, an extended Kalman filter (EKF) [39,40], and a second order polynomial Golay–Savitsky (GS) filter [41,42], which was established with a 17-point moving window. Low and high frequencies could be filtered with the GS filter by holding the maximum and minimum values [29]. The reference value of Δ*P* was set to 87 mmHg to meet the physiological perfusion of 5 L/min at rest, and *K_P_* and *K_I_* were 0.008 and 0.0016 for AFP and DP2, respectively. (4) Maintain a constant MAoP [30]. MAoP can be measured using a pressure sensor (sensor-based MAoP control). The reference MAoP was set as 100 mmHg to reach a total output of 5 L/min under rest condition. *K_P_* and *K_I_* were set to 0.007 and 0.0014, respectively, for both pumps. (5) Maintain an average LVEDP [31]. LVEDP can be measured using pressure sensors. The setpoint of LVEDP was 6.6 mmHg to match PF of 5 L/min under rest with *K_P_* = 0.045 and *K_I_* = 0.009 for both pumps. This sensor-based control method is referred to as the LVEDP control.

### 2.5. Simulation Description and Data Analysis

Several conditions were considered to quantify the overall performance of all the control algorithms: (1) rest and exercise; (2) rapid pulmonary vascular resistance (PVR) increase by eight-fold during rest and exercise; (3) rapid change in physiologic condition from rest to exercise and exercise to rest. Noise was included to simulate a uniformly distributed random variable up to ±5 to 10% of actual PF signals [27]. The simulated heart rates were 80 bpm during rest and 120 bpm during exercise. All the initial pump parameters were zero, approximating a realistic pump start condition.

MATLAB (MathWorks, Natick, MA, USA) was used for simulation, data reduction, and analysis including the calculation of PS, PF, CO, AoP, LVEDP, and left ventricular volume (LVV). The mean values in this simulation were calculated based on the final 20 cardiac cycles after the simulation reached a steady state. Instantaneous values of LV pressure less than 1 mmHg were considered to be a suction event [3,17,43].

## 3. Results

### 3.1. The Proposed SIMPF Control Algorithm

**Figure 2:**
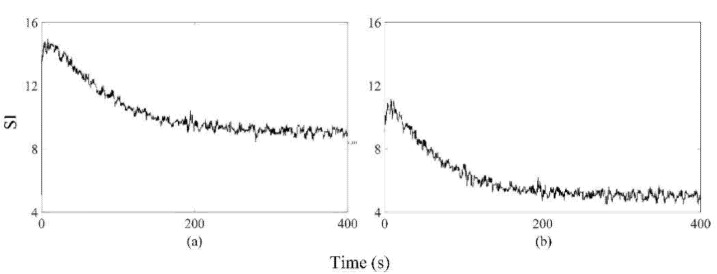
Measured pump flow signals were used to calculate *SI*, whose values gradually decreased and finally approached the setpoints of 9 for AFP (**a**) and 5 for DP2 (**b**), respectively.

**Figure 3:**
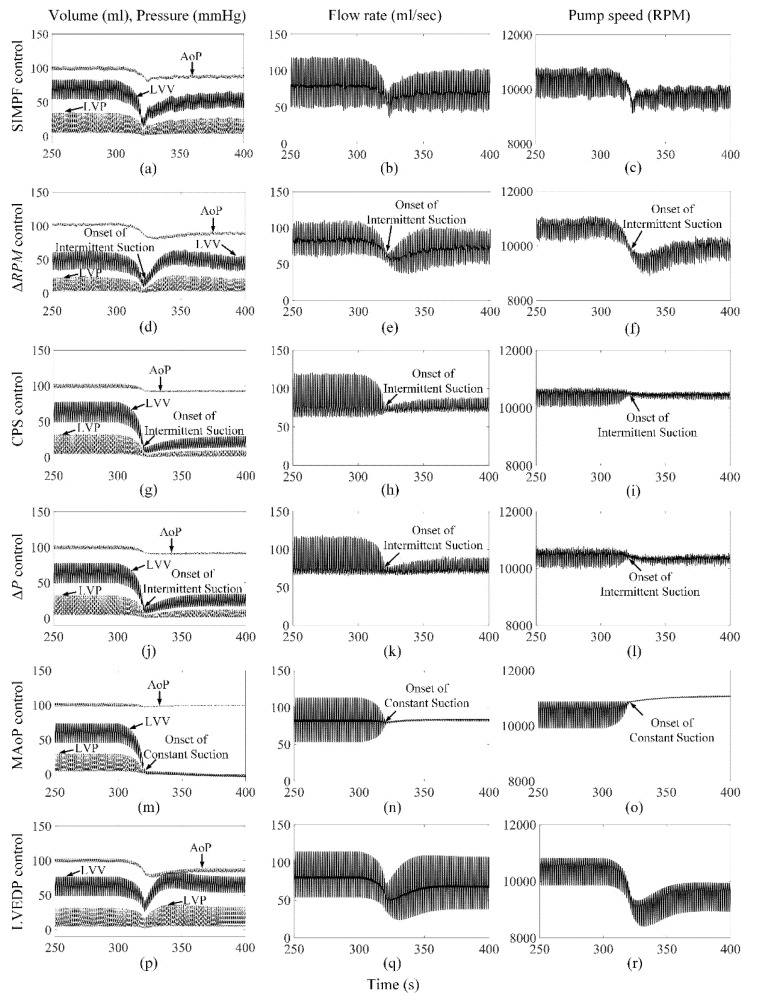
Comparison of performance among six control strategies at rest when the PVR increase eight times for AFP. The increase in PVR started when *t* = 300 s. (**a**−**c**) SIMPF control with 5% noise. (**d**−**f**) Δ*RPM* control. (**g**−**i**) CPS control. (**j**−**l**) Δ*P* control. (**m**−**o**) MAoP control. (**p**−**r**) LVEDP control. SIMPF and LVEDP control did not cause any LV suction. Δ*RPM*, CPS, and Δ*P* control induced intermittent suction. MAoP control induced constant suction.

**Figure 4:**
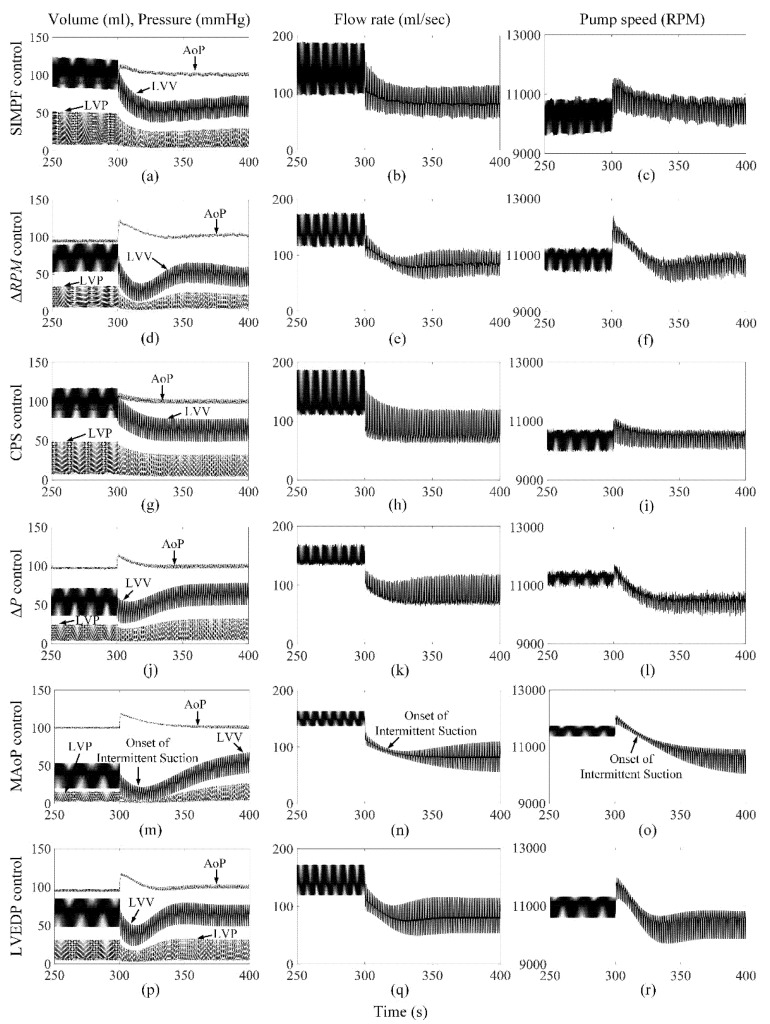
Comparison of performance among six control strategies under step change from exercise to rest for AFP. The transition started when *t* = 300 s. (**a**−**c**) SIMPF control with 5% noise. (**d**−**f**) Δ*RPM* control. (**g**−**i**) CPS control. (**j**−**l**) Δ*P* control. (**m**−**o**) MAoP control. (**p**−**r**) LVEDP control. Intermittent suction events were found for the MAoP control algorithm.

**Figure 5:**
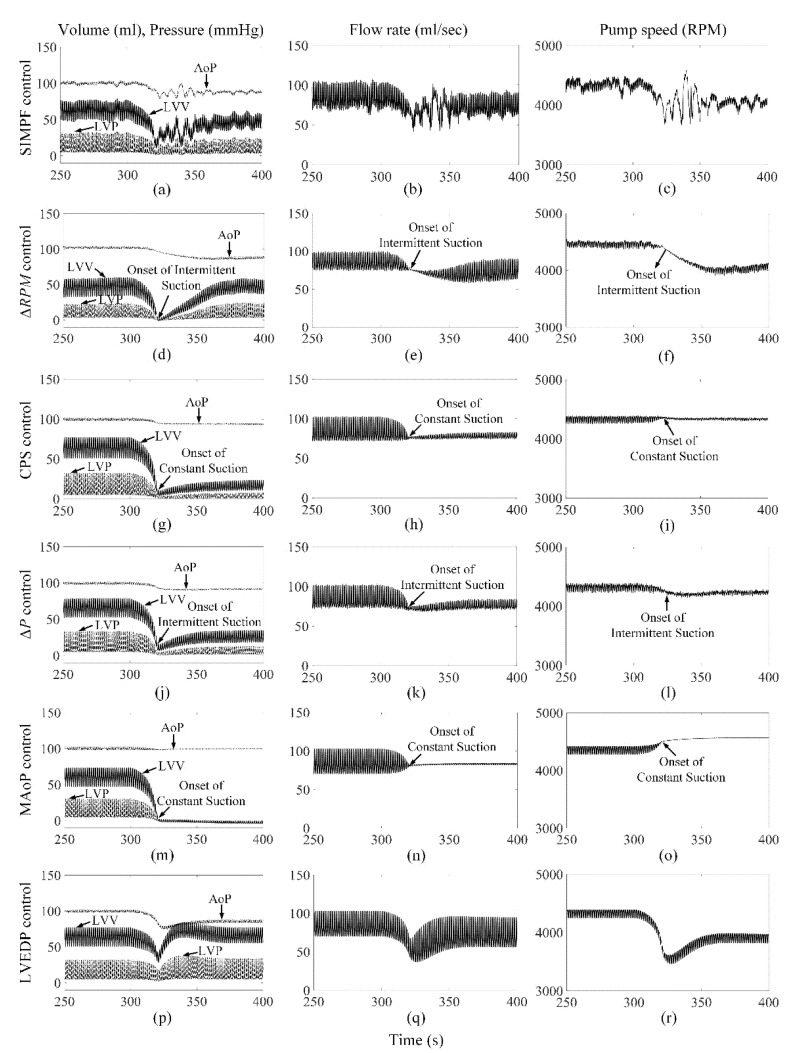
Comparison of performance among six control strategies at rest when the PVR increase eight times for DP2. The increase in PVR started when *t* = 300 s. (**a**−**c**) SIMPF control with 5% noise. (**d**−**f**) Δ*RPM* control. (**g**−**i**) CPS control. (**j**−**l**) Δ*P* control. (**m**−**o**) MAoP control. (**p**−**r**) LVEDP control. SIMPF and LVEDP control did not cause any LV suction. Δ*RPM* and Δ*P* control induced intermittent suction. CPS and MAoP control induced constant suction.

**Figure 6:**
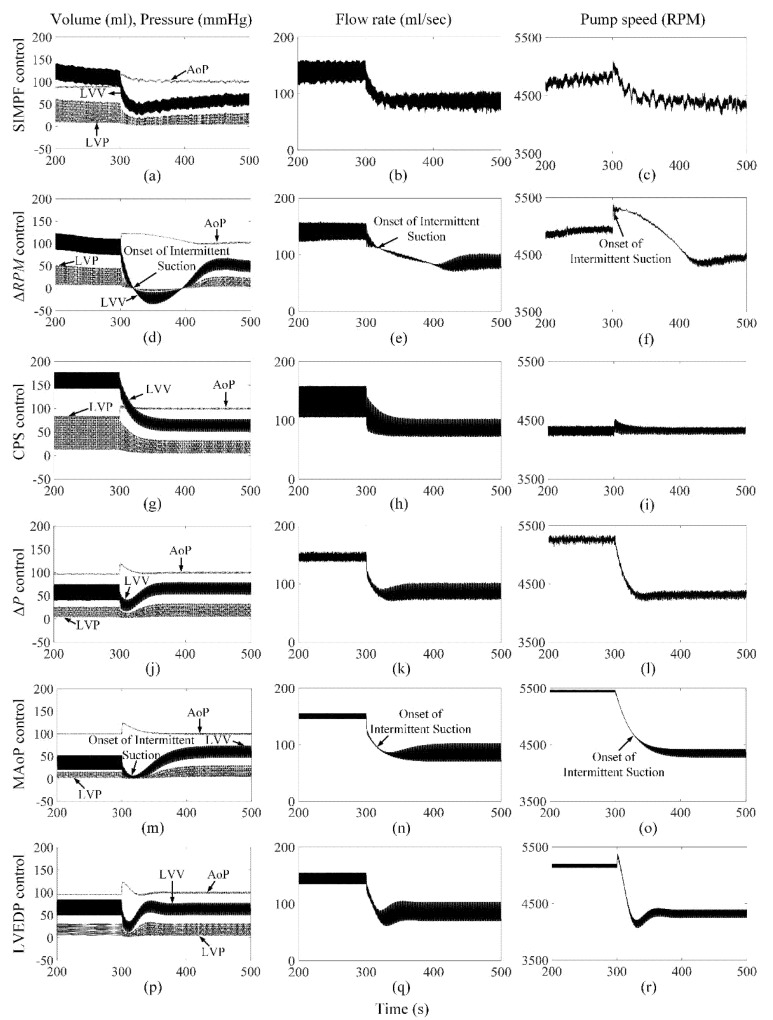
Comparison of performance among six control strategies under step change from exercise to rest for DP2. The transition started when *t* = 300 s. (**a**−**c**) SIMPF control with 5% noise. (**d**−**f**) Δ*RPM* control. (**g**−**i**) CPS control. (**j**−**l**) Δ*P* control. (**m**−**o**) MAoP control. (**p**−**r**) LVEDP control. Intermittent suction events were found for the Δ*RPM* and MAoP control algorithms.

Figure 2 shows the extracted *SI* from the measured pump flow signals for both axial and mixed flow pumps, respectively. The extracted *SI* was initially high at low LVAD flow rates and reduced gradually to 9 ± 0.5 and 5 ± 0.3, which were close to the *SIr* setpoints for AFP and DP2, respectively, while PF, PS, and control variable increased. The proposed SIMPF control algorithm provided sufficient physiologic perfusion and avoided suction during various conditions (Table 1 and Table 2). For AFP, the SIMPF control algorithm generated flow rates of 5 L/min and 8.2 L/min at rest and exercise (Table 1), respectively. Figure 3a–c and Figure 4a–c demonstrate that the SIMPF control strategy successfully avoided LV suction under conditions when the PVR increased 8-fold and during step change from exercise to rest for AFP. The simulated results of DP2 were similar to AFP during almost all test conditions (Figure 5a–c and Figure 6a–c). In addition, at rest, the root mean square errors (RMSE) of the measured values of pump flow rate were 2.4 mL/s (5% noise) and 4.8 mL/s (10% noise) for AFP, and 2.4 mL/s (5% noise) and 4.8 mL/s (10% noise) for DP2, respectively. At exercise, RMSE of the measured values of pump flow rate were 3.9 mL/s (5% noise) and 7.9 mL/s (10% noise) for AFP, and 3.8 mL/s (5% noise) and 7.9 mL/s (10% noise) for DP2, respectively.

### 3.2. ΔRPM Control Algorithm

The Δ*RPM* control algorithm successfully generated sufficient cardiac outputs and avoided LV suction events during rest and exercise conditions for both pumps (Table 1 and Table 2). However, as shown in Figure 3d–f and Figure 5d–f, an intermittent LV suction event occurred when the PVR was increased under rest, since the instantaneous LV pressure decreased to less than 1 mmHg. Meanwhile, no LV suction was found under exercise when the PVR was increased. The control algorithm could function effectively during the rapid step change from rest to exercise (no figures shown) and exercise to rest (Figure 4d–f) for AFP. However, for DP2, there was a serious intermittent LV suction event (~100 s), causing the minimum LVP to be −7.1 mmHg during the rapid transition from exercise to rest (Figure 6d–f).

### 3.3. CPS Control Algorithm

The CPS control strategy did not induce LV suction events without a change in PVR and provided adequate physiological perfusion during the rest condition (5.0 L/min) for both pumps. However, during exercise, the increase in pump flow was lower than that using any other control algorithm, especially for DP2 (Table 1 and Table 2). Figure 3g–i also shows that this control algorithm caused intermittent LV suction at rest with AFP when the PVR was octupled compared to the normal activities of the patients. It caused constant suction with DP2, as shown in Figure 5g–i. Furthermore, the CPS control strategy did not trigger LV suction events during rapid condition change from rest to exercise (no figure shown) and exercise to rest (Figure 4g–i and Figure 6g–i) and for both pumps.

### 3.4. ΔP Control Algorithm

Maintaining a fixed ΔP from LV to the aorta across AFP or DP2 provided physiological demands of 5.0 L/min and 8.7 L/min during rest and exercise conditions, respectively (Table 1 and Table 2). However, intermittent suction events were observed at rest and during exercise with a rapid 8-fold increase in PVR (Figure 3j–l and Figure 5j–i). No suction events were found during transitions between rest and exercise conditions.

### 3.5. MAoP Control Algorithm

For both pumps, the MAoP control algorithm guaranteed adequate end-organ perfusion at rest (5.0 L/min) and exercise (9.0 L/min) (Table 1 and Table 2). However, MAoP control failed to adapt to sufficient end-organ perfusion under rest (Figure 3m–o and Figure 5m–o) and exercise states when the PVR increased eight times, because the onset of constant suction was observed as the minimum value of LVP was negative. The controller also caused intermittent suction cases during the transition from exercise to rest (Figure 4m–o and Figure 6m–o).

### 3.6. LVEDP Control Algorithm

The LVEDP control strategy increased the pump flow rate from 5.0 L/min to 8.5 L/min for both pumps when the physiologic condition changed from rest to exercise (Table 1 and Table 2). No suction events were observed during all the tested conditions (Figure 3p–r, Figure 4p–r, Figure 5p–r and Figure 6p–r).

In summary, based on all the simulation results, the proposed control algorithm is pump-independent and outperformed other control strategies for both axial and mixed flow pumps by avoiding suction and providing physiologic levels of perfusion.

## 4. Discussion

The computer simulation results demonstrated the feasibility and performance of the proposed SIMPF control strategy to autonomously regulate pump flow rates. The human circulatory system has highly non-linear dynamics including flow discontinuities due to the presence of valves, and highly variable physiological perfusion needs. Similarly, the LVAD pump dynamics are non-linear and depend on the design of the pump. LVAD support to the circulatory system has several requirements and constraints, which makes the design of a control algorithm highly challenging: (1) LVADs have low preload and afterload sensitivity; (2) appropriate amounts of flow to meet varying cardiac demand must be maintained for the functional capacity of patients; and (3) over-pumping and suction need to be avoided even during the rapid reduction in preload (e.g., Valsalva). The proposed SIMPF algorithm adequately met the conflicting demands of maintaining perfusion demand and avoided LV suction events under various simulated conditions. Notably, the SIMPF control algorithm was effective at avoiding suction even when PVR was increased resulting in a drastic reduction in preload. Similarly, quick step transitions between exercise and rest were also simulated to produce conditions that are conducive to causing suction events. The proposed SIMPF control algorithm measured pump flow with 5–10 percent uniformly distributed noise added to the PF signals, which were used to extract *SI*. Up to 10% noise was used as this is the maximum error for ultrasonic flow probes used clinically.

Axial flow and mixed flow pumps are two different types of pumps. Axial pumps are based on the Archimedes screw, where the flow of the liquid is along the axis of the impeller. Mixed flow is a centrifugal flow pump with a mixed flow impeller. In the DP2 pump, the fluid experiences both radial acceleration and lift, and exits the impeller nearly perpendicular to the axial direction. There are differences in pump dynamics between the axial flow pump and DP2 pump, and different sensitivities between the two devices to the pressure head (HQ curves). However, the proposed SIMPF algorithm was able to achieve similar results using both AFP and DP2, demonstrating the pump-independence of the proposed control method. The *SI* values between the two pumps were notably different, as expected due to their intrinsic differences causing differences in pump flow used to extract *SI*, and also because pump types are affected differently by the contractility of the native heart. The SIMPF algorithm requires the direct measurement of flow. Measuring LVEDP using pressure sensors can provide similar performance compared to the SIMPF control strategy. However, pressure measurements are prone to failure and long-term drift due to contact with blood. A sensor drift of even 2–3 mmHg can cause significant performance degradation and suction with the LVEDP control algorithm. Unlike pressure sensors, ultrasonic flow probes have been successfully used for long term LVAD flow measurements. When mounted on the outflow graft of an LVAD, the performance of ultrasonic flow probes is not significantly affected by tissue ingrowth. Unfortunately, flow probe measurement noise is unavoidable, and control algorithm performance degrades with increasing measurement noise. However, even with 10% noise, the algorithm prevented suction and provided physiologic perfusion. The chosen measurement noise levels (5–10%) were based on the levels reported for ultrasound flow probes. The performance of the control algorithm can be further improved by periodic calibration of the flow probe.

The SIMPF algorithm requires some level of native LV function to generate the variation in flow rate for *SI*. This native ventricular contractility is always present clinically. If ventricular asystole occurs, it would also result in the loss of right ventricular function and lead to mortality, even in the presence of adequate LVAD support. During exercise, an increase in preload due to venous return will increase native ventricular contractility due to the Frank–Starling mechanism. This increase in contractility will increase *SI*, which in turn will result in increased LVAD flow to meet the perfusion demand. Similarly, a reduction in LV contractility caused by reduced physiological demand would generate lower pump flow rates to prevent LV suction. A 5 s moving window was used in this study to minimize oscillations in *SI* values due to varying heart rates that are independent of physiological conditions. A shorter moving window will increase the speed of response, but may be more sensitive to noise and transient events.

The performance of the proposed SIMPF control strategy was superior compared to previously proposed control algorithms. It provided physiologically relevant cardiac outputs that were similar to other algorithms proposed in the literature, but was better at avoiding suction compared to the other sensor-based and sensorless control algorithms, especially when elevated PVR occurred briefly during the Valsalva maneuver or coughing. Rapid and sustained PVR increase by eight-fold during rest and exercise does not occur in nature. This was done to demonstrate the robustness of the controller to avoid suction even under non-physiologic, extreme conditions. Furthermore, sensor based control algorithms are usually model-based and predict pressure heads and flows. They usually require *a priori* knowledge of the blood viscosity or can be erroneous in the case of inflow or outflow kinking or thrombus formation in the blood pump. While incorporating a flow sensor increases complexity, an actual measurement of flow obviates the need for estimation and improves the performance of the controller under inflow/outflow graft kinks or thrombus formation that occurs in patients implanted with LVAD. The flow probes that were previously implanted with DeBakey/Heart Assist 5 LVADs underwent rigorous durability testing prior to approval by the Food and Drug Administration and no flow probe failures have been reported during LVAD implants.

In this study, the in silico computer simulation model provided a meaningful initial step for early tested hypotheses, but it cannot replace mock flow loop studies, animal testing, and clinical trials. For instance, it cannot fully replicate the complex in vivo dynamics including tissue remodeling, autonomous regulation, and neurohumoral responses. The lumped parameter human circulatory system model has several inherent limitations due to assumptions of ideal heart valves, Newtonian blood, and ignores the effects of gravity and inertia. However, the in silico model demonstrated the feasibility control algorithms despite these limitations. Pre-clinical in vitro and in vivo experiments will be used to validate the SIMPF control algorithm.

## 5. Conclusions

A new flow sensor-based SIMPF control strategy was developed for rotary LVADs to provide adequate cardiac output and prevent LV suction. This proposed control strategy implemented the control objective using an effective *SI* extracted from the measured pump flow signal. Two different types of rotary LVADs were incorporated to quantify the performance of the SIMPF strategy, showing promising results. This algorithm is pump-independent and can be incorporated into existing LVAD control systems.

## Figures and Tables

**Table 1:** Performance comparison among the proposed SIMPF control strategy and other control algorithms during various test conditions with AFP.

	CO (L/min)	AoP (mmHg)	Min LVP (mmHg)	LVV (mL)	Mean PS (RPM)	Suction
Healthy heart without LVAD support						
Rest	5.0	122/80	2.7	43/106	N/A	No
Exercise	8.6	121/74	2.8	42/114	N/A	No
HF without LVAD support						
Rest	3.8	97/63	15.5	181/229	N/A	No
Exercise	6.8	95/58	15.4	178/234	N/A	No
HF with AFP support at rest						
SIMPF control ^1^	4.9	102/97	4.7	54/82	10,365	No
SIMPF control ^2^	5.0	103/98	4.4	49/77	10,441	No
Δ*RPM* control	5.1	104/101	3.3	35/62	10,717	No
CPS control	5.0	103/97	4.4	49/78	10,448	No
Δ*P* control	5.0	103/97	4.4	50/78	10,447	No
MAoP control	5.0	103/99	4.1	46/74	10,506	No
LVEDP control	5.0	103/98	4.4	49/77	10,449	No
HF with AFP support at exercise						
SIMPF control ^1^	8.1	94/89	6.7	75/106	10,505	No
SIMPF control ^2^	8.2	95/91	6.1	68/98	10,653	No
Δ*RPM* control	8.5	97/94	4.8	51/80	11,000	No
CPS control	8.1	94/88	7.0	79/109	10,450	No
Δ*P* control	8.7	99/96	3.5	36/64	11,276	No
MAoP control	9.0	101/99	2.2	20/47	11,590	No
LVEDP control	8.5	98/94	4.5	48/77	11,037	No
HF with AFP support during 8-fold increase in PVR at rest						
SIMPF control ^1^	4.3	90/85	1.2	46/68	9712	No
SIMPF control ^2^	4.3	90/86	1.1	42/64	9800	No
Δ*RPM* control	4.4	90/87	0.8	37/58	9900	IS
CPS control	4.6	94/92	0.5	14/29	10,448	IS
Δ*P* control	4.6	93/91	0.6	17/34	10,355	IS
MAoP control	5.0	100/100	−1.8	−2/−1	11,065	CS
LVEDP control	4.2	89/84	2.3	54/77	9547	No
HF with AFP support during 8-fold increase in PVR at exercise						
SIMPF control ^1^	7.1	83/78	2.7	65/90	9867	No
SIMPF control ^2^	7.2	84/79	2.3	58/83	10,013	No
Δ*RPM* control	7.3	85/81	1.4	51/75	10,176	No
CPS control	7.5	86/83	2.2	38/60	10,450	No
Δ*P* control	8.1	92/90	0.5	13/26	11,149	IS
MAoP control	8.9	100/100	−5.1	−13/−7	11,950	CS
LVEDP control	7.3	84/80	1.7	53/77	10,125	No

^1^ With 5% noise. ^2^ With 10% noise.

**Table 2:** Performance comparison among the proposed SIMPF control strategy and other control algorithms during various test conditions with DP2.

	CO (L/min)	AoP (mmHg)	Min LVP (mmHg)	LVV (mL)	Mean PS (RPM)	Suction
Healthy heart without LVAD support						
Rest	5.0	122/80	2.7	43/106	N/A	No
Exercise	8.6	121/74	2.8	42/114	N/A	No
HF without LVAD support						
Rest	3.8	97/63	15.5	181/229	N/A	No
Exercise	6.8	95/58	15.4	178/234	N/A	No
HF with DP2 support at rest						
SIMPF control ^1^	5.0	102/98	4.2	50/76	4337	No
SIMPF control ^2^	5.0	103/100	3.0	38/63	4419	No
Δ*RPM* control	5.1	104/101	3.2	35/60	4385	No
CPS control	5.0	102/98	4.5	51/77	4336	No
Δ*P* control	5.0	101/98	4.6	53/78	4326	No
MAoP control	5.0	102/99	4.2	47/74	4363	No
LVEDP control	5.0	102/98	4.5	51/77	4339	No
HF with DP2 support at exercise						
SIMPF control ^1^	8.0	92/89	7.4	85/112	4856	No
SIMPF control ^2^	8.3	94/92	5.8	66/93	5023	No
Δ*RPM* control	8.1	92/90	6.9	78/105	4957	No
CPS control	7.3	84/80	12.0	142/169	4337	No
Δ*P* control	8.7	98/96	3.8	40/67	5268	No
MAoP control	9.0	101/100	2.3	21/47	5453	No
LVEDP control	8.5	96/94	4.6	50/77	5173	No
HF with DP2 support during 8-fold increase in PVR at rest						
SIMPF control ^1^	4.3	89/86	1.0	41/62	4048	No
SIMPF control ^2^	4.4	91/88	0.5	32/51	4104	IS
Δ*RPM* control	4.4	90/87	−0.4	36/56	4067	IS
CPS control	4.7	95/93	0.3	11/24	4336	CS
Δ*P* control	4.6	93/91	0.6	18/34	4243	IS
MAoP control	5.0	100/100	−1.8	−3/−1	4566	CS
LVEDP control	4.2	88/84	2.4	56/77	3911	No
HF with DP2 support during 8-fold increase in PVR at exercise						
SIMPF control ^1^	7.1	81/78	3.0	70/92	4462	No
SIMPF control ^2^	7.3	83/81	2.0	54/76	4616	No
Δ*RPM* control	6.9	79/76	1.5	82/105	4340	No
CPS control	6.9	79/76	5.9	83/106	4337	No
Δ*P* control	8.1	91/90	0.5	15/28	5101	IS
MAoP control	8.9	100/100	−5.1	−13/−8	5572	CS
LVEDP control	7.3	83/81	1.5	55/77	4604	No

^1^ With 5% noise. ^2^ With 10% noise.

## Data Availability

Data are made available through the corresponding author upon a reasonable request.
